# Nanocellulose: A Fundamental Material for Science and Technology Applications

**DOI:** 10.3390/molecules27228032

**Published:** 2022-11-19

**Authors:** Aiswarya Poulose, Jyotishkumar Parameswaranpillai, Jinu Jacob George, Jineesh Ayippadath Gopi, Senthilkumar Krishnasamy, Midhun Dominic C. D., Nishar Hameed, Nisa V. Salim, Sabarish Radoor, Natalia Sienkiewicz

**Affiliations:** 1 Department of Polymer Science and Rubber Technology, CUSAT, Kochi 682022, India; 2 Department of Science, Faculty of Science & Technology, Alliance University, Chandapura-Anekal Main Road, Bengaluru 562106, India; 3 Department of Mechanical Engineering, PSG Institute of Technology and Applied Research, Coimbatore 641062, India; 4 Department of Chemistry, Sacred Heart College (Autonomous), Kochi 682013, India; 5 Department of Mechanical and Product Design Engineering, Swinburne University of Technology, Hawthorn, Victoria 3122, Australia; 6 Faculty of Science, Engineering and Technology, Swinburne University of Technology, Victoria 3122, Australia; 7 Department of Polymer-Nano Science and Technology, Jeonbuk National University, 567 Baekje-daero, Deokjin-gu, Jeonju-si 54896, Republic of Korea; 8 Faculty of Chemistry, Institute of Polymer and Dye Technology, Lodz University of Technology, Stefanowskiego 16, 90-537 Lodz, Poland

**Keywords:** nanocellulose, biodegradable, composites, applications

## Abstract

Recently, considerable interest has been focused on developing greener and biodegradable materials due to growing environmental concerns. Owing to their low cost, biodegradability, and good mechanical properties, plant fibers have substituted synthetic fibers in the preparation of composites. However, the poor interfacial adhesion due to the hydrophilic nature and high-water absorption limits the use of plant fibers as a reinforcing agent in polymer matrices. The hydrophilic nature of the plant fibers can be overcome by chemical treatments. Cellulose the most abundant natural polymer obtained from sources such as plants, wood, and bacteria has gained wider attention these days. Different methods, such as mechanical, chemical, and chemical treatments in combination with mechanical treatments, have been adopted by researchers for the extraction of cellulose from plants, bacteria, algae, etc. Cellulose nanocrystals (CNC), cellulose nanofibrils (CNF), and microcrystalline cellulose (MCC) have been extracted and used for different applications such as food packaging, water purification, drug delivery, and in composites. In this review, updated information on the methods of isolation of nanocellulose, classification, characterization, and application of nanocellulose has been highlighted. The characteristics and the current status of cellulose-based fiber-reinforced polymer composites in the industry have also been discussed in detail.

## 1. Introduction

Increasing ecological concerns and the depletion of fossil fuels have led to a continuously growing demand for the development of sustainable and environmentally friendly biocomposites [1,2,3,4,5,6,7]. A biocompatible composite can be obtained by perfectly blending the finest properties of a biodegradable polymer and natural filler [8]. Fiber reinforcement ingrained with polymers is effective in enhancing the properties of the composites. These composites have the advantage of both the polymers and the fibers. To enhance the mechanical properties of the composites an efficient load transfer should be established between the polymer and fiber [9]. The performance of the composite depends upon the properties of the fiber and the polymer, fiber geometry and orientation, fiber dispersion, and fiber volume fraction [10]. Therefore, many studies are focused on the modification of fibers and their uniform distribution in the polymer matrix. 

The demand for biofibers as reinforcing fillers has intensified recently due to their unique properties such as low density, low cost, biodegradability, ease of availability, recyclability, good acoustic and thermal insulation, low irritation to the skin, etc. [11,12]. It is a strategic approach to replace traditional carbon and glass fibers with plant fibers in the advanced composite industry. Plant fibers have numerous potentialities, they can create rural jobs, have good physio-mechanical properties, decrease energy consumption, and also one can do lots of chemistry on the surface of plant fibers. Ample research on the isolation of plant fibers with different length scales was reported [13,14,15,16,17,18]. Among the different length scales nanocellulose is preferred due to the high crystallinity, high modulus (ca. 130 GP), good tensile strength, low density (ca. 1.5 g/cm^2^), reactive surface, and high-water holding capacity [19,20]. It is worth adding that the nanocellulose is the strongest part of the plant, present in the cell wall. The polymer chains in nanocellulose are mostly in a perfectly crystalline state. Therefore, they are very strong and stiff [21]. 

**Figure 1:**
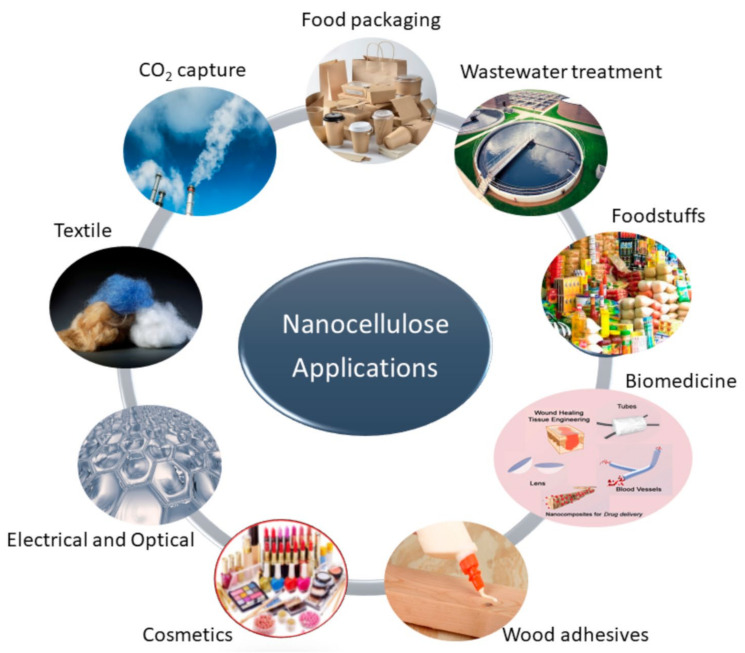
Important applications of nanocellulose [30]. (Open access).

Several methods (usually in combinations) such as grinding [22], high-pressure homogenization [22,23], cryocrushing [24,25], steam explosion [26], ultrasonication [27], enzymatic pre-treatments [28,29], and chemical treatments [15,16,17] are used for the isolation of nanocellulose from plant fibers. However, mechanical treatments are energy consuming; therefore, chemical or chemo-mechanical treatment methods of cellulose sources are mostly preferred [20,25,26]. Some of the important applications of nanocellulose are given in Figure 1 [30]. In this review, chemical treatment methods are discussed in detail. This review summarizes the chemical structure of cellulose, the classification of nanocellulose based on their size, source, methods employed for extraction, and applications. The fabrication, properties, and applications of cellulose-based fiber-reinforced polymer composites have also been discussed in detail.

## 2. Chemical Structure of Cellulose

Cellulose has a chemical formula (C_6_H_10_O_5_)_n_. The degree of polymerization (n) varies between 10,000 and 15,000 depending upon the source [31]. It is a homopolysaccharide made up of β-1, 4 anhydro-D-glucopyranose units [32]. The pyranose structure of glucose is made up of six carbon atoms. The glucose units are linked by the acetal bonds (bonds formed through single oxygen) between the C_1_ carbon of one pyranose unit and the C_4_ carbon of another pyranose unit [33]. 

**Figure 2:**
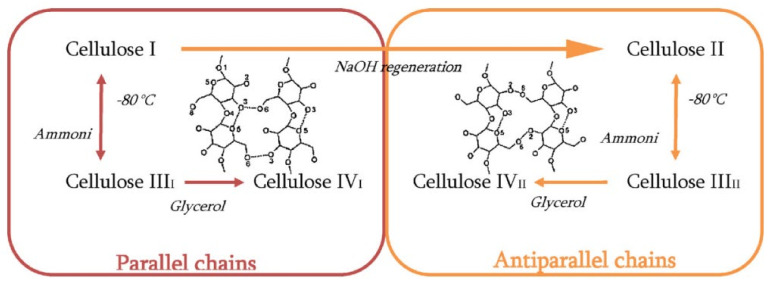
Methods to obtain different cellulose polymorphs. Reprinted/adapted with permission from Ref. [38]. License Number: 5413150701249.

Cellulose exists in four polymorphs, cellulose I, II, III, and IV, depending on the source, treatments, and methods of extraction. The most studied celluloses are cellulose I and cellulose II [34]. Cellulose I or native cellulose is the naturally occurring cellulose, which is crystalline and thermodynamically metastable. Cellulose I coexist as two polymorphs Iα and Iβ. Algae and bacterial cellulose consist of Iα, while Iβ is dominated in higher plants. The Iα cellulose has a triclinic unit cell with one cellulose per unit cell and can be converted to Iβ by hydrothermal treatments in an alkaline solution. Cellulose Iβ has a monoclinic unit cell with two cellulose chains per unit cell. The cellulose chains in both Iα and Iβ have a parallel arrangement such that the β (1, 4) glycosidic bonds orient in the same direction [35,36]. Cellulose II allomorph rarely occurs in nature and is thermodynamically more stable than cellulose I. Cellulose II has a monoclinic unit cell, with chains in antiparallel confirmation, and can be produced from cellulose I by regeneration and mercerization, and the transition between cellulose I and cellulose II is irreversible [37]. Cellulose III is reactive crystalline cellulose that exists in two forms, III _I_ and III _II_. It can be produced from cellulose I and cellulose II by treatment with ammonia. The modification of cellulose III with glycerol produces cellulose IV [38]. The methods to obtain different polymorphs of cellulose are shown in Figure 2. 

## 3. Source of Nanocellulose

**Figure 3:**
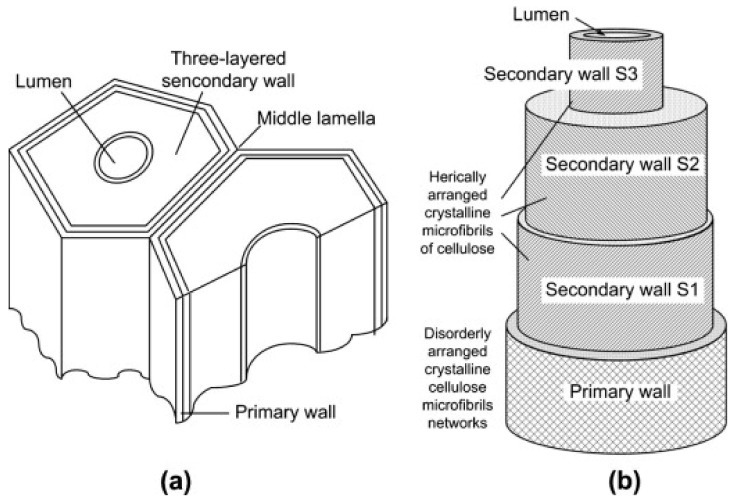
Structure of natural fibers (**a**) cell wall (**b**) structural components. Reprinted with permission from Ref. [45]. License Number: 5413150388892.

Cellulose is the most abundant natural polymer extracted mainly from woody and nonwoody residues, tunicates, bacteria, and algae [39,40]. The most abundant cellulose source is woody and nonwoody residues [41,42,43,44]. A schematic of the structure of plant fiber is shown in Figure 3 [45]. The plant fiber consists of an exterior primary cell wall, followed by three secondary walls and a lumen at the center. The primary cell wall contains disorderly arranged cellulose fibers while the secondary cell wall contains helically arranged cellulose fibers. The cellulose fibers provide strength to the plant fibers. Other sources such as tunicates, bacteria, and algae are also used to produce cellulose. Tunicates found in oceans are a rich source of cellulose. The cellulose in tunicates is similar to plant cellulose and mostly exists in the Iβ form of cellulose [40]. Bacterial cellulose is produced by bacteria and *Acetobacter xylinum* (or *Gluconacetobacter xylinus*) is the most efficient bacteria to produce cellulose. The features of bacteria cellulose are high crystallinity, elastic modulus, and degree of polymerization [46]. Several algal taxa such as *Chlorophyta*, *Charophyceae*, *Rhodophyta*, *Phaeophyceae*, and *Dinophyta* are found to have cellulose in their cell wall and the cellulose mostly exists in Iα form [47].

## 4. Classification of Nanocellulose

**Figure 4:**
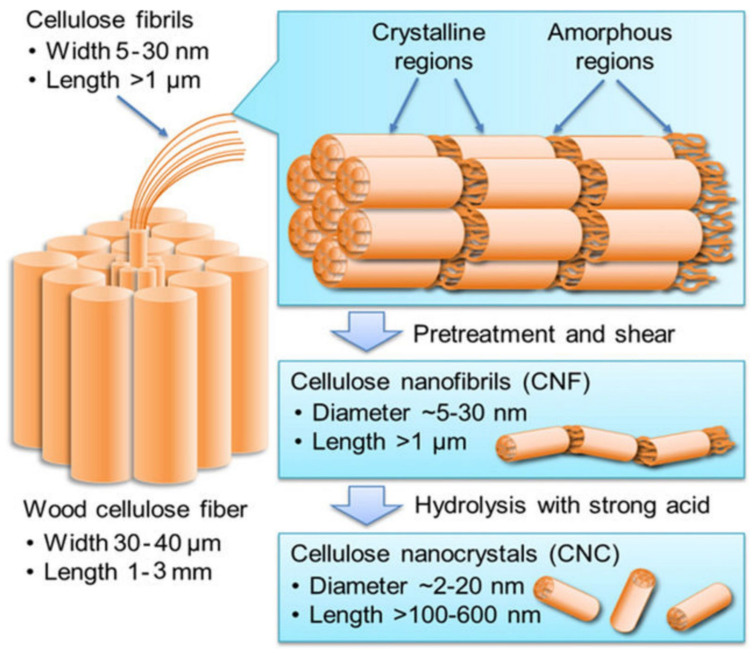
A schematic of production of the NFC and CNC from wood. Reprinted with permission from Ref. [49]. Copyright © 2016, American Chemical Society.

Nanocellulose is generally classified as cellulose nanocrystal (CNC), nanofibrillated cellulose (NFC), or cellulose nanofibrils (CNF) based on their dimensions, functions, preparation methods employed, and the source of production [48]. A schematic of the production of the NFC and CNC from wood is shown in Figure 4 [49]. The first step is the retting process to make microfibrils, followed by pre-treatments, and shearing to produce CNF. The CNF on strong acid hydrolysis gives cellulose nanocrystals.

### 4.1. Nanofibrillated Cellulose (NFC)

**Figure 5:**
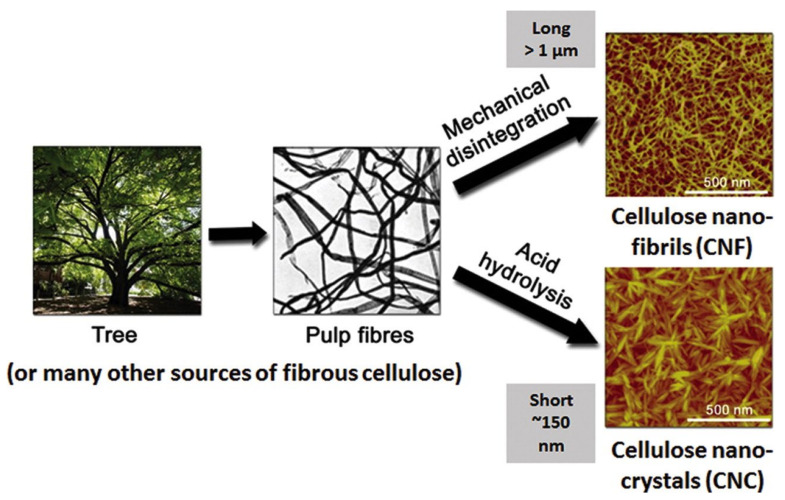
Isolation of CNF and CNC from cellulose source [52]. (Open access).

Nanofibrillated cellulose or cellulose nanofibrils are flexible fibers that consist of alternating crystalline and amorphous domains with a high aspect ratio, diameter ranging from 5–30 nm, and length of several micrometers [49,50]. NFC can be extracted from a broad range of sources such as plants, animals, and bacteria. The dimensions, crystallinity, and morphology of the obtained fibrillated cellulose depend mainly on the mechanical treatments and the raw natural sources [51]. It is usually extracted by enzymatic or chemical (pretreatments) methods followed by mechanical treatments. The enzymatic and chemical methods are used to remove the hemicellulose and lignin contents while the mechanical method is employed to reduce the size of microfibrils into nano dimensions. Cationic and anionic charges are introduced on the surface of the cellulose fibrils as a result of pre-treatments. This helps to reduce high energy consumption during mechanical treatment and also helps to stabilize the fibrils. Carboxymethylation, carbonylation, alkali treatment, acid hydrolysis, sulfonation, and quaternization are some of the chemical pre-treatments used. The mechanical treatment induces high shearing forces on the cellulose bundles, which helps in defibrillation [50,51,52,53,54]. The various mechanical treatments used for nanofibrillation are high-pressure homogenization, microfluidization, micro grinding, steam explosion, cryo-crushing, and high-intensity ultrasonication [51]. Figure 5 shows the isolation of CNF from cellulose sources [52].

### 4.2. Cellulose Nanocrystal (CNC)

Cellulose nanocrystals can be isolated from different cellulosic sources such as plants, animals, bacteria, and algae. These are highly crystalline forms of cellulose, which contain 100% cellulose and the crystallinity ranged between 54 and 88%. It possesses stiffness, a high aspect ratio, high specific strength, surface area, and unique liquid crystalline properties. Moreover, the CNC is rich in Iβ crystal structure (68–94%) [31]. They are rod-like whisker-shaped particles with a width range between 2 and 70 nm and a length from 100 to 600 nm [49,52,55]. 

The cellulose nanocrystals are usually isolated by the pre-treatment of cellulose sources for partial removal of hemicellulose and lignin content followed by acid hydrolysis (Figure 5) [49] or enzymatic treatment of the cellulose fibers [56]. The acid treatment helps in the removal of the amorphous sections in the cellulose fibers and increases the crystalline content. It usually requires harsh reaction conditions and lesser time than enzymatic hydrolysis. The mineral acids used in acid hydrolysis determine the surface functionalities of the nanocellulose. The nanocellulose produced from sulphuric acid possesses high colloidal stability due to the presence of negatively charged sulphate groups surrounding the cellulose chains. The repulsive nature of these surface groups prevents further agglomeration of the cellulose chains.

The structure and dimension of the nanocellulose depend widely on (i) the cellulose source (ii) the concentration of mineral acid (iii) the duration of treatment and (iv) the temperature of hydrolysis [57]. The characteristics of the cellulose nanocrystals mainly depend on the presence of hydroxyl groups present on their surface, surface area, aspect ratio, crystallinity, and mechanical properties [58]. The surface hydroxyl groups serve as active centers for hydrogen bonding with the polar matrix. The hydroxyl groups are highly reactive and therefore can be modified by surface chemical functionalization, which improves the compatibility between the non-polar polymer matrix and the nanocellulose. The strong inter- and intra-chain hydrogen bonding offers strength, stiffness, and crystallinity to the cellulose nanocrystals. Therefore, their incorporation in the polymer may enhance the thermo-mechanical performance of the polymer matrix.

## 5. Pre-Treatments of Cellulose Sources

The efficient conversion of lignocellulosic biomass into pure cellulose occurs through different steps. Pre-treatment is the primary method adopted for the breakdown of cell walls and internal tissues of the lignocellulosic biomass through biochemical conversion processes [59]. This treatment disrupts internal structures and opens a channel for further treatments. Hence the cellulose sources are susceptible to size reduction, alteration of structure, increasing crystallinity, and improving the swelling capacity in water during the pre-treatments [60].

The pre-treatments are classified into physical, biological, and chemical methods. Physical pre-treatment includes mechanical, pyrolysis, sonication, microwave radiation, and spray drying [61]. Grinding, chipping, and milling are the different processes that are usually adopted as mechanical pre-treatments. Chipping is performed to reduce the particle size to several millimeters. While the high shearing forces produced by milling and grinding methods provide uniform size distribution and reduce the particle size to 0.2 mm. The specific surface area, degree of polymerization, and crystallinity of the nanocellulose depend upon the type and duration of the milling methods employed [62]. Vibratory milling, wet disk milling, and dry milling are the different types of milling methods used to reduce the size of plant fibers. Mechanical treatment is a good option as a pre-treatment because no toxic by-products are produced during the process and are economically viable. The biological process includes bacterial treatment, enzymatic treatment, the action of fungi, and picking. The biological treatments lead to hemicellulose and lignin decomposition [61].

The main chemical pre-treatments adopted for cellulose extraction includes alkali pre-treatment and bleaching [61,62]. The alkali treatment followed by bleaching is the primary strategy adopted in the purification of cellulose. This method removes a portion of lignin, wax, hemicellulose, and pectin that surrounds the cellulose in the cell wall. Alkali treatment persuades ionization of hydroxyl groups of cellulose into alkoxide and thus reducing the number of hydroxyl groups on the cellulose surface [63]. Thus, the surface roughness and hydrophobicity of the fiber increase, resulting in the adhesion of cellulose molecules with the polymer matrix. The different chemical treatments involved in the extraction of nanocellulose from plant fibers such as pre-treatments (alkali treatment and bleaching), acid treatments, treatments with ionic liquids, and deep eutectic solvents are discussed as follows.

### 5.1. Alkali Treatment

**Figure 6:**
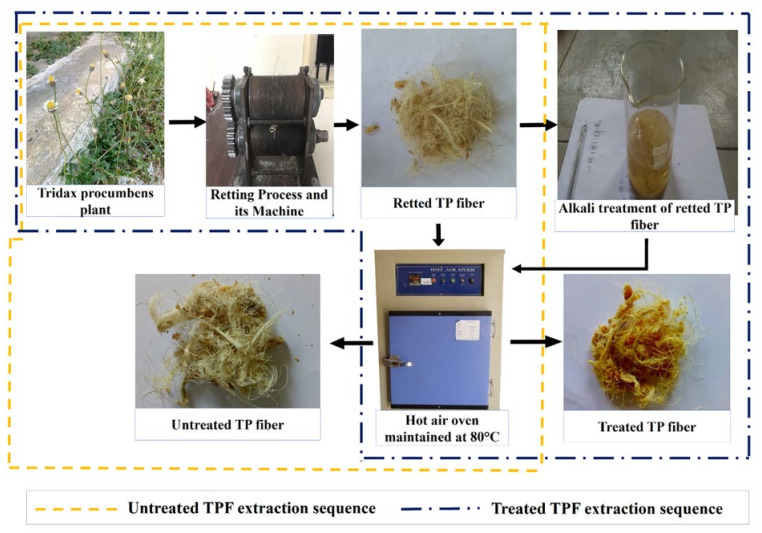
Pictorial representation of alkali treatment of *Tridax procumbens* plant. Reprinted with permission from Ref. [66]. License Number: 5424600342017.

The phenomenon of alkali treatment involves the solvation and decomposition of lignocellulose components. Alkali solution penetrates into the crystallites to destroy the inter and intra molecular hydrogen bonds in the cellulose molecules and the neighboring crystalline region. Amorphous fraction such as hemicellulose is not densely packed as the crystalline region of cellulose and hence provides more accessibility for alkali penetration [64]. The degree of crystallinity and crystalline index is increased by the removal of these amorphous components. Moreover, the removal of lignocellulosic components especially hemicellulose causes the interfibrillar region of the fibers to become less dense and rigid. Due to the removal of hemicellulose, the fibrils can rearrange themselves along the direction of tensile deformation which promotes even load distribution in the fibers [65]. Alkali treatment is also effective in the partial removal of lignin from the fiber [66]. A pictorial representation of the alkali treatment of the Tridax procumbens plant is shown in Figure 6 [66].

The alkali treatment is usually carried out under room temperature conditions; hence it is a less energy-intensive treatment compared to other pre-treatment methods [67]. The advantages of the alkali treatment method include low operation cost and minimal degradation of cellulose sources [61,68,69]. The main reagents used in alkali treatment include hydroxides of sodium, potassium, calcium, and ammonium, of which sodium hydroxide was found as most effective [70]. Costa et al. [71] treated corn stover with a 2% sodium hydroxide solution to remove the impurities and waxy substances covering the external surface of the fibers. The alkali treatment swells the fiber structure, which increases the surface area and thereby enhances the ease of hydrolysis of cellulose polymer chains. Wulandari et al. [72] extracted nanocellulose fibers from sugarcane bagasse by pre-treating the source with 17.5% NaOH solution for 3 h at 45 °C with constant stirring to remove the hemicellulose content.

**Figure 7:**
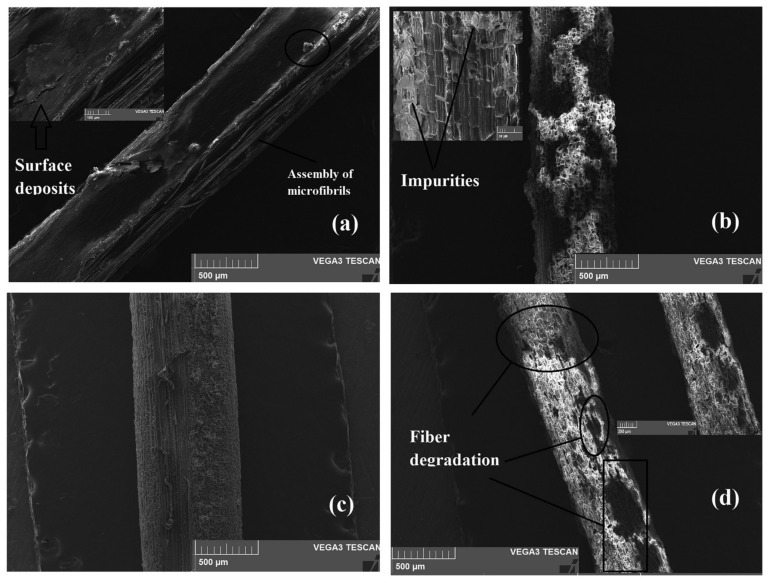
SEM micrographs showing the effect of NaOH treatment on date palm fiber texture compared to raw fiber: (**a**) raw fiber, (**b**) 2% alkali treated, (**c**) 5% alkali treated and (**d**) 10% alkali treated [75]. (Open access).

Movva et al. [73] reported partial removal of the non-cellulosic components from pistachio shells using alkali treatment. However, the binding material lignin remains intact. Different concentrations of sodium hydroxide with different soaking times were used by Jayabal and his co-workers [74] for the removal of non-cellulosic components from coir fiber. From the scanning electron micrographs, the researchers confirmed the partial removal of non-cellulosic components from coir fiber during alkali treatment. Oushabi et al. [75] studied the effect of alkali treatment on the mechanical, morphological, and thermal properties of date palm fibers by different percentages of sodium hydroxide. The fibers were treated with 2, 5, and, 10% of NaOH for 1 h at 25 °C. The 5% alkali-treated fibers showed the best tensile and flexural strength due to the removal of nano-cellulosic components present in the fibers. However, at higher concentrations of alkali (10%), fiber degradation occurs as observed from the SEM micrographs (Figure 7), this will degrade the properties of the fiber. The thermal stability of the 5% alkali-treated fiber was also better when compared with pure date palm fibers. Thus, the best results were observed at 5% alkali treatment.

To avoid the wastage of toxic alkali and for better removal of amorphous components of natural fibers, recently many researchers are focused on alkali treatment combined with the steam explosion method, microwave-assisted methods, enzymatic pre-treatments, and hot water treatment. Cellulose fibers have been extracted from jute fibers by treating them with a 2% sodium hydroxide solution followed by a steam explosion. A portion of hemicellulose and lignin components were removed during the alkalization, while further removal occurs during steam explosion because of depolymerization and defibrillation [76]. Ouyang and co-workers [77] extracted the cellulose fibers from corncob using the steam explosion and alkali treatment simultaneously. The results of this experiment showed a synergistic effect on the removal of lignin and hemicellulose with less time and lower alkali consumption. Kim et al. [78] used a percolation reactor, which comprises two stages, such as (i) hot water treatment and (ii) treatment with ammonia for the fractionation of corn stover. During the hot water treatment, hemicellulose was removed, while the ammonia treatment easily removed the lignin content. Thus, the effective removal of non-cellulosic materials is possible with this method. Kaur and co-workers [69] analyzed the structural changes in paddy straw after the alkali and enzymatic pre-treatment. The paddy straw was treated with different alkalis such as NH_3_, Na_2_SO_3_, Na_2_CO_3,_ and NaOH, followed by microwave treatment. The microwave treatment works on the heat transfer mechanism and helps to degrade lignin components. Their studies confirmed that the microwave-assisted NaOH pre-treatment is the most efficient one, which decreases the lignin and silica content to a considerable extent. 

### 5.2. Oxidation/Bleaching Treatment

**Figure 8:**
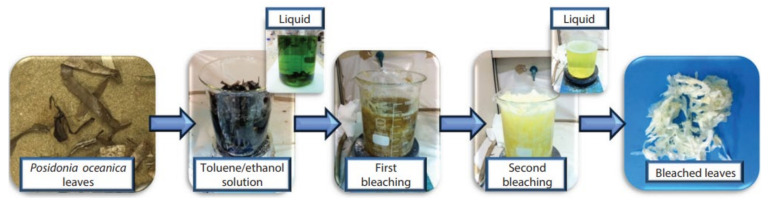
Pretreatment of *Posidonia oceanica* leaves [82]. (Open access).

The alkali-treated fibers were further subjected to bleaching. Bleaching removes lignin from the lignocellulosic fibers. As stated above the alkali treatment remove only the lignin components partially; therefore, delignification is essential for the effective defibrillation of cellulose fibers [17,79]. Moreover, the presence of lignin on the cellulose components decreases the wettability between the polymer matrix and the natural fiber. Sodium chlorite (NaClO_2_), sodium hypochlorite (NaClO), hydrogen peroxide (H_2_O_2,_), and sodium sulfite (Na_2_SO_3_) are the main bleaching agents used in the delignification of cellulose fibers [58,80]. The presence of lignin imparts a brown color to the cellulose material, which fades during the delignification process. As the delignification progresses the bleaching solution turns to bright yellow color and the cellulose material into white [81]. Luzi et al. [82] used two bleaching steps for the removal of lignin components from *Posidonia oceanica* leaves treated with toluene/ethanol solution (Figure 8). The fibers were first bleached with sodium chlorite followed by acetic acid and later with 5% sodium bisulphate (NaHSO_4_). The whitening of the leaves after the two bleaching treatments confirms the complete removal of the lignin. 

Abdel-Halim et al. [83] used sodium chlorite, triethanolamine salts (activating agent), and Texazym T (non-ionic wetting) agent for bleaching alkali-treated olive tree branch. This method provides an efficient route for delignification by elevating the oxidation potential of the bleaching agent. The FTIR confirmed the removal of lignin after the bleaching. Ng et al. [58] reviewed that in an acidic medium, sodium chlorite is capable of liberating chlorine dioxide (ClO_2_), which oxidizes the lignin by attacking the aromatic region. This helps to solubilize the lignin and the fibrillation of the cellulose. Sá et al. [84] used sodium hypochlorite (2.5%) and a buffer solution (1:1 acetic acid (5%) and sodium hydroxide (5%)) for 2 h at 80 °C as the bleaching agent for the successful elimination of lignin. The fibers were washed several times with water and dried in an oven at 70 °C after bleaching. The IR spectrum of the sample shows that a C-H group characterizing the lignin disappeared after the bleaching treatment. 

**Figure 9:**
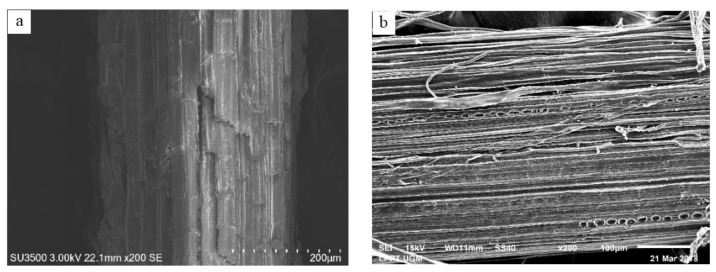
SEM images of (**a**) untreated salacca midrib fibers (**b**) bleached salacca midrib fibers [89]. (Open access).

The delignification process may produce harmful byproducts and are not environmentally friendly. Rehman et al. [85] adopted chlorine-free bleaching of alkali-treated eucalyptus lenceolata for the isolation of cellulose. The pulp was first treated with the hydrogen peroxide solution and ethylenediaminetetraacetate (EDTA), followed by CH_3_COOH and HNO_3_ solution. In an interesting work, Wu et al. [80] successfully used more environmentally friendly hydrogen peroxide as a bleaching agent and citrate dehydrate as a pH stabilizer. Duan et al. [86] used peracetic acid for the bleaching of the jute fibers. The researchers used FTIR to confirm the complete removal of lignin after the peracetic acid treatment, due to the reaction of peracetic acid with lignin in the jute fiber. Moreover, the crystallinity of the fibers was increased due to the dissolution of the amorphous region during the treatment. Grumo et al. [87] used a solution made up of sodium chlorite and acetate buffer as a bleaching agent for the removal of lignin from alkali-treated pineapple leaves. The bleaching treatment was performed at 100 °C for 4 h. The researchers were able to produce high-quality cellulose fibers by alkali + bleached treatment. Mussatto and his coworkers [88] extracted cellulose pulp from Brewers Spent Grain (BSG) by chlorine-free bleaching using hydrogen peroxide. The initial bleaching was performed using 5% hydrogen peroxide stirring at 70 °C for 40 min. The process was repeated twice, followed by alkali treatment with 5% sodium hydroxide. Yudha and his coworkers [89] bleached alkali-treated salacca midrib fibers using 3% of hydrogen peroxide. The SEM images of untreated and bleached fibers are given in Figure 9. After the chemical treatment, the microfibers were formed by defibrillation of cellulose fibers which is evident from the SEM images.

## 6. Acid Hydrolysis

For the successful extraction of nanocellulose, selective removal of the amorphous region is inevitable. Among several chemical and biological treatments, acid hydrolysis is the most convenient procedure adopted for obtaining pure cellulose materials [48,58]. Due to structural imperfections, the amorphous region of cellulose is more susceptible to acid hydrolysis than the crystalline region. That means the glycosidic bond breakage in the amorphous region is faster than it occurs in the crystalline region. The acid hydrolysis depends on the factors such as concentration of acid, duration of the treatment, temperature of the reaction, etc. A detailed understanding of the mechanism of acid treatment is essential for the extraction of nanocellulose materials, having desired morphology and structure [58,61]. Generally, optimum acid hydrolysis occurs only at high temperature and high pressure. However, if the temperature is more than 110 °C, then highly toxic compounds such as furfural and 5-hydroxymethyl furfural will evolve [61]. Both mineral acids and organic acids are used for acid hydrolysis.

### 6.1. Mineral Acid Hydrolysis

The primary mineral acids, such as sulphuric acid, hydrochloric acid, hydrobromic acid, phosphoric acid, etc., are used in the hydrolysis of treated fibers. The advantages of mineral acids in the isolation of nanocellulose are a few processing steps, high cellulose yield, high crystallinity, good thermal stability, small size, and reproducibility. On the other hand, they are toxic and corrosive. Amongst the mineral acids, sulphuric acid is the most common acid used in hydrolysis treatment [58,90]. The sulphuric acid not only reduces the hemicellulose and lignin components but also introduces sulphate groups on the surface of the cellulose materials. The electrostatic dispersion of negatively charged sulphate groups provides colloidal stability to the cellulose material when dispersed in water [91]. In general, pre-treated cellulose fibers were treated with 60–68% of concentrated sulfuric acid for 1 to 2 h at 40–65 °C to obtain effective hydrolysis [48,91,92].

Punnadiyil and his co-workers [91] isolated microcrystalline cellulose (MCC) from peanut shells using sulphuric acid. The FTIR absorption band at 1735 cm^−1^ and 1247 cm^−1^ corresponds to the C=O stretching vibration of the acetyl group in hemicellulose and lignin, which disappeared after the acid hydrolysis. In an interesting study, Mohamed et al. [92] successfully isolated rod-like nanocellulose crystals from pre-treated recycled newspaper using H_2_SO_4_. The generated cellulose nanocellulose has a length of ca. 121 nm, and the yield of cellulose nanocrystals prepared was 54.6% with a crystallinity index of 90%. The thermal stability of the cellulose nanocrystals drops due to the presence of sulphate groups. Campano et al. [93] later reported the extraction of cellulose nanocrystals from old newspapers and recycled newsprint using sulphuric acid hydrolysis with good CNC purity of approximately 76 to 78%. Plermjai et al. [94] reported the successful extraction of nanocellulose from sugarcane bagasse using the ball milling-assisted acid hydrolysis method. The FTIR and XRD results support the removal of amorphous components from the cellulose fibers. The UV spectra of nanocellulose showed greater UV absorption due to the small size and large surface area of the extracted cellulose. Maaloul and his co-workers [95] extracted cellulose nanocrystals from almond shells using sulphuric acid hydrolysis using a dialysis-free method. The XRD results showed the highest crystallinity value for the acid-hydrolyzed cellulose fibers compared to pre-treated and untreated fibers, indicating the dissolution of the amorphous region from the acid-hydrolyzed cellulose fibers. Thus, studies revealed that sulphuric acid hydrolysis is one of the most effective methods for the extraction of nanocellulose; however, there are several drawbacks that limit its use such as corrosion to the reactor, environmental incompatibility, and reduced thermal stability [55,96]. 

Hydrochloric acid (HCl) is also widely used for the extraction of cellulose nanocrystals from plant fibers [56]. Chandra and her coworkers [97] extracted cellulose nanofibrils from areca nut husk fibers using alkali pretreatment, followed by hydrochloric acid hydrolysis and bleaching. The study relived that the pretreatment followed by acid hydrolysis removed the unwanted amorphous components from cellulose. The initial raw fiber reported 34% cellulose content in the areca nut husk fibers, while the HCl-treated composites reported 67.78% cellulose content. After bleaching with NaClO_2_/CH_3_COOH, the% cellulose content increases to 85.47%. The FTIR, XRD, SEM, FESEM, TEM, and DLS support the dissolution of amorphous parts from the fibers during the acid treatment. The TGA showed enhanced thermal stability for the nanocrystals formed due to the increased crystallinity. For raw fiber, the% crystallinity was 37%, while that of acid-treated fibers was 60%. Hastuti et al. [98] studied the extraction of CNC from oil palm empty fruit bunches (bleached kraft OPEFB pulp) using HCl. The extracted CNCs were further characterized by elemental analysis, electron microscopy, XRD, birefringence observation, and thermal analysis. The results revealed the isolation of CNC from oil palm empty fruit bunches using HCl hydrolysis. The thermal stability of the CNC also reported a high value due to the high crystallinity. Many studies reported sedimentation of CNC suspension, prepared by HCl hydrolysis. However, the researchers found that the CNC suspension remained stable even for 6 months with clear birefringence, showing nano dispersibility.

In another interesting work, Chen et al. [99] used a two-step treatment, nitric acid-ethanol pretreatment and treatment in a fiber disintegrator for the extraction of cellulose from poplar wood. The samples were treated multiple times. After three treatment cycles ca. 95.6% lignin and 63.8% hemicellulose were removed. The obtained cellulose was dissolved in ionic liquid and cellulose films were prepared. The films showed a tensile strength of 32.8 MPa, elongation at break of 47.5%, and transmittance of 80%. There was a drop in the mechanical properties of the cellulose films prepared after the fourth treatment cycle, possibly due to the degradation of the cellulose caused by the excess hydrolysis of cellulose because of the treatment with nitric acid. Hanani et al. [100] studied the effect of various acids on the extraction of microcrystalline cellulose from rice husk (RH). The rice husk was first pre-treated with alkali, followed by bleaching before the acid hydrolysis. The researchers used three different acids, HNO_3_, H_2_SO_4_, and HCl for hydrolysis. The MCC obtained after the chemical treatments were confirmed by the FTIR results. The yield percentage of MCC-RH obtained after the treatment was 83.5, 80.6, and 81.8 for HNO_3_, H_2_SO_4_, and HCl, respectively. The crystallinity of the MCC obtained from XRD studies was 54.2, 52.4, and 49.7 for HNO_3_, H_2_SO_4_, and HCl, respectively. Thus, the study showed the suitability of using HNO_3_ for the successful extraction of MCC from RH. Trifol et al. [101] chemically extracted nanocellulose with stable dispersion from pre-treated sisal fibers using acetic and nitric acid. EM, XRD, and FTIR confirmed the removal of amorphous components after the chemical treatments. The yield of the CNF was 38.9%. Other than toxic chemicals, less toxic environmentally friendly materials such as phosphotungstic acid can be used. Lu et al. [102] used a mechanochemical method for the extraction of nanocellulose from bamboo pulp. The bamboo pulp and 13.5 wt% phosphotungstic acid were added to the agate jar and ball milled. The yield of CNC was 88.4%, and the characteristic properties such as XRD, TGA, FTIR, TEM, and TGA confirmed the isolation of CNC with high crystallinity and thermal stability.

### 6.2. Organic Acid Hydrolysis

The advantages of organic acid in the isolation of nanocellulose are less toxicity, good yield, thermal stability, crystallinity, and less corrosion. Therefore, in recent years, many research studies are focused on using organic acids for extracting cellulose from raw natural fibers. However, for effective isolation of nanocellulose high temperature and more reaction time are required [58,103,104]. Erdogan et al. [104] successfully used pre-treated banana fibers for the extraction of cellulose using formic acid. In another work, Nazir et al. [105] extracted cellulose from oil palm empty fruit bunches using formic acid and hydrogen peroxide. The yield of the extracted cellulose was 64%, with 90% alpha-cellulose content and 70% crystallinity. In an interesting work, Yeganeh et al. [106] used two different acids maleic acid and sulfuric acid for the extraction of cellulose from waste office paper. It was observed that the cellulose extracted using maleic acid showed higher crystallinity, yield, and dispersion compared to the cellulose extracted using sulfuric acid. This is due to the degradation of the cellulose caused by the strong sulfuric acid.

## 7. Treatment Using Ionic Liquids (ILs)

**Figure 10:**
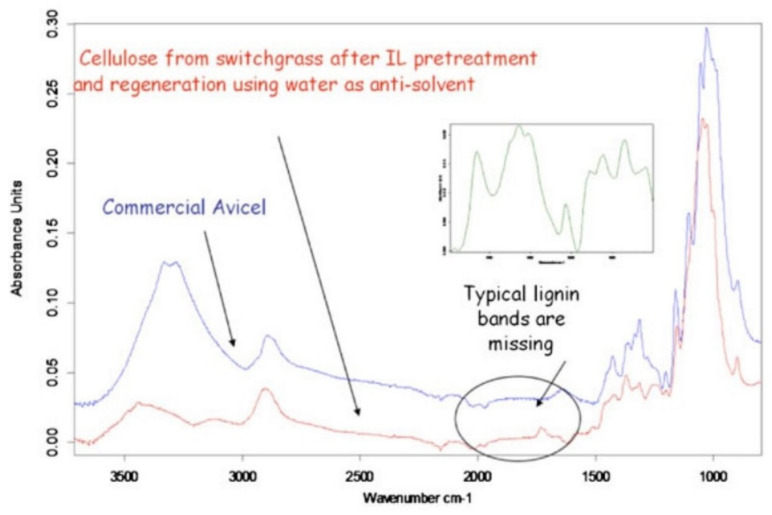
The FTIR spectra of the regenerated cellulose using water. Reprinted with permission from Ref. [111]. License Number: 5413151287587.

Ionic liquids are green solvents and are widely used recently for the extraction of cellulose from plant fibers [107,108]. Ionic liquids are organic salts made up of cations and anions having melting points less than 100 °C. The cations are generally organic moieties such as imidazolium while the anions can be chloride, acetate, and hydrogen sulfate. The presence of both anions and cations plays an important role in solubilizing or swelling the cellulose network structure. The ionic liquids can form hydrogen bonds with the -OH groups on the cellulose surfaces resulting in the dissolution of intra- and inter-molecular hydrogen bonds between the cellulose molecules [109]. Here the -OH group in cellulose molecules forms an electron donor—the cations in IL act as an electron acceptor, thus a complex is formed with the interaction of cellulose with ILs [110]. Later, the cellulose can be regenerated from the ionic liquid by the addition of nonsolvents such as water or ethanol. Singh et al. [111] reported the dissolution of switchgrass using 1-n-ethyl-3-methylimidazolium acetate. The researchers regenerated the cellulose with the addition of water and observed that most of the lignin present in the fiber remains solubilized in IL as shown in FTIR spectra (Figure 10). Thus, IL will increase the crystallinity index and stiffness of the extracted cellulose.

**Figure 11:**
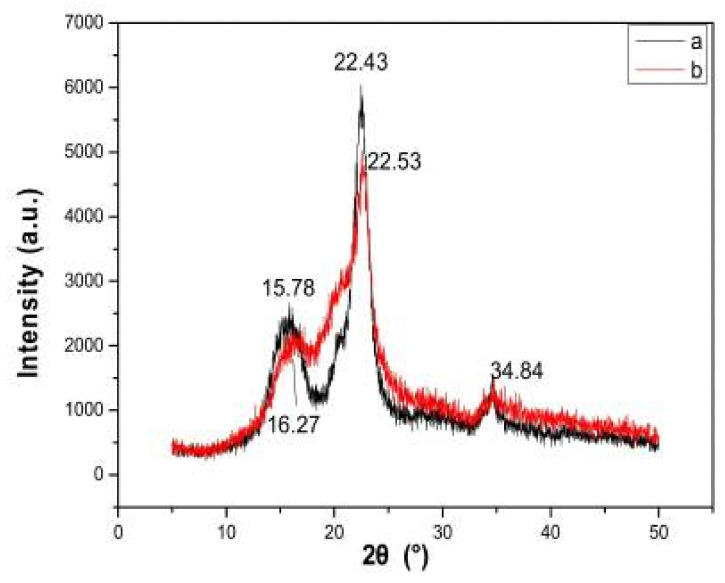
XRD pattern of (**a**) virgin cellulose, (**b**) regenerated cellulose [113]. (Open access).

Chowdhury et al. [112] extracted cellulose nanowhiskers (CNWs) from alkali-treated and bleached dried leaves of *Adansoia kilima* using ultrasonication in the presence of 1-butyl-3-methylimidazolium hydrogen sulfate (BmimHSO_4_). The interaction between the IL and treated dried leaves resulted in the formation of CNW crystals. Recently, deep eutectic solvents (DES) were also utilized for the modification of natural fibers and were more preferred than IL due to their less toxicity, biodegradability, and low cost. Zhang et al. [113] studied the solubility of cellulose in four eutectic solvents such as glycerol/chlorocholine (CHCl), citric acid/CHCl, urea/CHCl, and oxalic acid/CHCl. Oxalic acid/CHCl exhibited the best dissolution due to the increased hydrogen bonding interaction between the eutectic solvent and cellulose. The XRD pattern (Figure 11) showed that the virgin cellulose and regenerated cellulose have a similar structure.

## 8. Post Treatments

The used acid in the cellulose suspension was neutralized using post-treatment methods such as dialysis, ultrasonication, vacuum filtration, centrifugation, and neutralization by adding NaOH and H_2_O [114,115,116]. Mujtaba et al. [115] successfully synthesized cellulose nanocrystals from flax fibers using HCl. The obtained CNC were dialyzed to neutral and sonicated for dispersion, later filtered using the vacuum filtration method, and collected in a Petri dish. Orasugh et al. [116] isolated cellulose nanofibrils from jute using sulphuric acid hydrolysis. The nanocellulose suspension was neutralized by water washing using centrifugation, followed by drying using the freeze-drying method.

The isolated cellulose in aqueous suspension has a greater tendency for agglomeration due to the hydrophilic groups present in it. Therefore, to maintain the nano-dimensions of nanocellulose, drying processes such as supercritical drying, freeze-drying, oven drying, and spray drying are used [117,118]. Among the different methods, oven drying is the least preferred because the heat generated during the oven drying caused the agglomeration of the nanocellulose [58]. Lyophilization or freeze drying is a simple but powerful technique to remove water from nanocellulose suspension. Lyophilization consists of two steps: (1) freezing, and (2) drying by sublimation. The freezing involves the formation of ice crystals and the sublimation involves the evaporation of ice crystals and any non-frozen bond water molecules. The direct sublimation of the ice crystals prevents the agglomeration of nanocellulose. However, some studies reported agglomeration of nanocellulose during the freeze-drying process [58,117,118,119].

Another method that enables to obtain nanoscale dimensions of dried nanocellulose was supercritical drying. In this method, the water is replaced by the solvent exchange process using ethanol and then with liquid CO_2_ under supercritical conditions. However, the water in the CNC suspension cannot be completely replaced by this method. This method is suitable only for the laboratory scale and not for the industrial scale [58]. The most commonly used drying method of cellulose is spray drying. During the spray drying, the solids dispersed in the fluid system are sprayed on a hot plate as fine droplets resulting in the formation of powdered samples, pellets, or granules. Spray drying is a continuous process having low operation costs. When the number of solids in the suspension increases, viscosity also increases. This results in the formation of large droplets and, finally, an increase in particle size [58,117,120]. Among the different methods, Peng et al. [117] suggest spray drying as an efficient method for the drying of nanocellulose suspensions.

## 9. Current Reports on Nanocellulose and Their Applications

Due to its exceptional properties, nanocellulose is widely used in tissue engineering, nanomedicine, biosensors, biodegradable polymers, power storage, water treatment, etc. [121]. Vitamin D_3_ is essential for strong bones, and muscles, however significant fraction of the global population had low levels of vitamin D_3_ which resulted in weak bones, weak muscles, bone pain, etc. Colturato et al. [122] in his study used nanocellulose films to overcome this issue, in their work nanocellulose was used as a drug delivery system for the controlled release of vitamin D_3_. The nanocellulose films were cast using cotton linter and Vitamin D_3_. FTIR studies reported no interaction or no new bond formation between nanocellulose and vitamin D3. Further, the FTIR and SEM studies confirmed the presence of vitamin D_3_ in the cellulose sample. The rate of release of vitamin D_3_ was evaluated using UV–vis spectroscopy and observed a release of 3029 IU mL^−1^ of vitamin D_3_ in 1 h into the receptor liquid. The researcher recommends the modified cellulose membrane as a new model for vitamin supplementation. 

**Figure 12:**
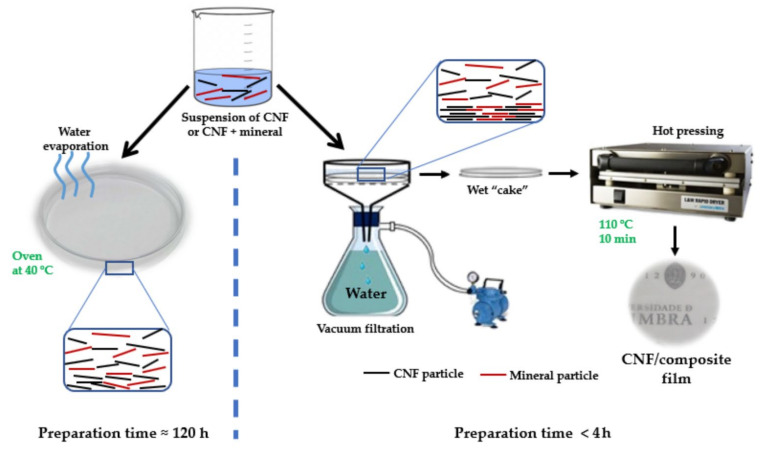
Schematic of fabrication of CNF/clay composite films via solvent casting and vacuum filtration/hot-pressing method [123]. (Open access).

Alves et al. [123] isolated CNF from eucalyptus globulus kraft pulp by three different methods, (i) high-pressure homogenization (mechanical treatment), (ii) enzymatic followed by mechanical treatment, and (iii) TEMPO oxidation followed by mechanical treatment. The researchers combined isolated nanocellulose with clay (sepiolite) and fabricated films with either solvent casting method or by vacuum filtration followed by hot pressing as shown in Figure 12. The films prepared by filtration + hot pressing provide superior tensile strength and Young’s modulus compared to solvent casting. The researcher proposes fabricated films as the replacement for plastics.

**Figure 13:**
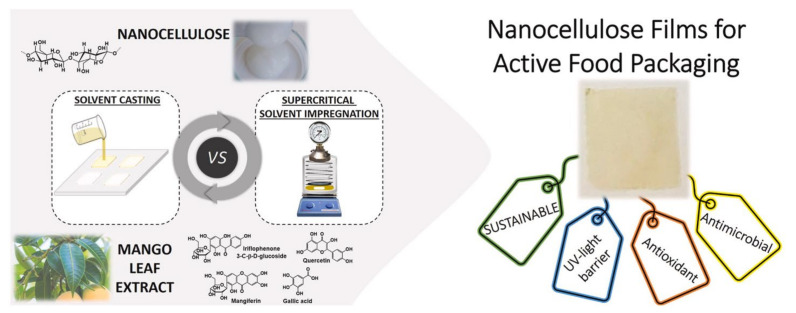
Schematic of the preparation of nanocellulose films via solvent casting and super-critical impregnation technology. Reprinted with permission from Ref. [126]. License Number: 5413160314308.

Al-Hagar and Abol-Fotouh [124] synthesized bacterial cellulose (*from Komagataeibacter hansenii KO28*) by a cost-effective method. In this method, the researchers used different doses of gamma radiation on the bacteria strain. The bacteria strain exposed to gamma radiation of 0.5 kGy twice (low dose) gives the maximum yield, i.e., 475% higher than the control culture after 10 days of incubation. Further, the synthesized nanocellulose was characterized and the properties agree with the control nanocellulose. Thus, the researchers believe that the outcome of this work could be a turning point in the synthesis of bacterial cellulose in larger amounts at a relatively lower cost. Nanocellulose was extracted from water hyacinth using acid hydrolysis [125]. The extracted nanocellulose was treated with NaOH and urea to form hydrogels. Later the hydrogels were treated with borax to form crosslinked hydrogels. The SEM morphology of the crosslinked nanocellulose showed a more porous structure. The uncross-linked hydrogels showed a swelling ratio of 325.2%, while the borax crosslinked cellulose hydrogel reported a swelling ratio of ~ 900% due to the increased OH groups on the crosslinked nanocellulose generated by the borax. The water content and gel fraction were also increased in the crosslinked nanocellulose, this is due to the increased OH groups and a higher degree of polymerization in the nanocellulose respectively. The crosslinked hydrogels also reported antibacterial activity against Gram-positive bacteria (*S. aureus*) with reasonably good thermal stability (main thermal degradation occurs between 250 and 400 °C) and transmittance. Thus, a super adsorbent hydrogel from nanocellulose was synthesized with possible application in wound dressing, agricultural, and flame-retardant coating. Bastante et al. [126] fabricated bioactive films with nanocellulose and mango leaf extract. The bioactive films were prepared by solvent casting or by super-critical impregnation technology. The method of preparation of nanocellulose films is schematically shown in Figure 13. The mango leaf extract has properties such as it is antioxidant and antimicrobial, therefore the nanocellulose films prepared showed antimicrobial properties against *Staphylococcus aureus* (Gram-positive) and *Escherichia coli* (Gram-negative). Apart from antioxidant and antimicrobial properties, the films prepared by super-critical impregnation technology reported good UV-light barrier properties and hence provide better preservation of the food for longer times. 

The wastewater generated from domestic sewage, and industries are causing serious problems for biota and the environment. Dyes are the main pollutant from the textile industry, there are several methods to remove dyes from water bodies such as coagulation, biological treatment, membrane filtration, and adsorption. But these methods are not favorable because they are not fully effective and also not fully green [127]. Recently researchers have been working on replacing toxic dyes using biomaterials such as cellulose or modified forms of cellulose [128,129,130]. Abdelaziz et al. [131] isolated nanocrystals from wastepaper (using acid hydrolysis) and used the nanocrystals for the preparation of hydrogels using epichlorohydrin as a crosslinker. The nanocrystal hydrogels are positively charged at lower pH and are negatively charged at higher pH. The selective adsorption of dye with cellulose is based on the electrostatic interaction. Therefore, the researchers reported excellent adsorption of acid red 8 anionic dye (due to the presence of sulfonic groups) at lower pH, but on the other hand, the dye will be desorbed at higher pH (alkali washing). The reusability of the hydrogels was also studied, and the dye removal percentage was reduced to half after four cycles, due to the degradation of films due to alkali washing. The kinetic parameters of the adsorption of the dyes were also studied, pseudo-second-order and Langmuir isotherm models fitted well with experimental data.

Nanocellulose is extensively used in medical applications such as drug carriers. Therefore, the distribution of nanocellulose in cells and tissues should be tracked. Babi et al. [132] successfully labeled fluorescently the nanocellulose using triazine-alkyne linker and azide dyes, without affecting the cellulose structure. The labeling was optimized and the labeled nanocellulose was identified using high-resolution fluorescence microscopy. Shishehbor and co-workers [133] studied the effect of fiber length, fiber orientation, fiber–fiber interaction, and loading direction on the mechanical properties of cellulose nanocrystals (CNC) films. The researchers used a coarse-grained model to study the effect of the above-mentioned parameters on the mechanical properties. Interestingly, the researchers observed that it is the interfacial strength between the cellulose nanocrystals (fiber–fiber interaction) is the main factor controlling the mechanical properties. Heidarian et al. [134] synthesized 3D printable self-healing hydrogels from carboxyl methyl chitosan, oxidized cellulose nanofibers, and chitin nanofibers. The imine crosslinks created between the nanomaterials caused the generation of nanohybrid hydrogels. The nanohybrid hydrogels were further treated with tannic acid (TA) and Fe III solution to impart conductivity. The gel showed good self-thinning properties, up to 89% self-healing, 100% self-recovery (without external stimuli), and good strain-sensing ability. 

Uetani et al. [135] reported a thermal conductivity of 2.5 W/m K for tunicate nanocellulose sheets, this value is more than other plastic films used in electronic devices. Thus, the nanocellulose films could be used as the base material for flexible electronic devices to prevent overheating. Fu et al. [136] fabricated cellulose-based flexible electronics from Balsa wood (*Ochroma pyramidale*). The balsa wood was chemically treated to remove the hemicellulose and lignin and later pressed with heavy loads to obtain a flexible transparent wood film with good strength and modulus. The electron-spun lignin was carbonized into a conductive carbon fiber before making it into an amyloid/lignin-based carbon ink. The ink was then printed on the flexible transparent wood film to develop an electronic circuit. The researchers claim that the developed flexible electronics were environmentally friendly, sustainable, and recommend for applications such as sensors and flexible circuits. Yuen et al. [137] fabricated ultra-thin biosensors by using nanocellulose from bacteria. The circuit boards were successfully mounted on the nanocellulose by ink-jet printing and electroless plating. The researchers recommend the developed nanocellulose-based circuit boards for health care, electronics, and temperature sensor applications. Carter et al. [138] developed a CNF manufacturing plant to produce repeatable CNF slurry by using mechanical defibrillation technology. Later CNF sheets were fabricated from CNF slurry either by calendar or non-calendar methods. The thickness was minimum for calendared sheets; hence, the calendared sheets are more transparent. The researchers reported changes in the transparency of the films by altering the solids content of the slurry or by changing the height of the knife applicator. The nanosheets were further sterilized (using ethylene oxide) for applying it as animal implants (mice and non-human primates). It was observed that the nanosheet implants were fully biocompatible with animals with no irritation or inflammation. The other applications and challenges of nanocellulose such as inhibiting bacterial growth in plants, bone regeneration, and treatment of bones were also reported [139,140].

## 10. Fabrication Methods of Polymer Composites

There are several methods available for the fabrication of nanocomposites. The important methods are in situ polymerization, solvent casting, melt intercalation, extrusion, and injection molding [141,142,143,144]. In the in-situ polymerization method, the monomer is mixed with the nanofiller in the presence of an initiator. Later the monomer is polymerized. Solvent casting is the simplest of all methods and is the most widely used approach for the fabrication of composites. Here the polymer is dissolved in a suitable solvent and after proper dissolution, the filler is incorporated into the solution and cast in a casting chamber. After the complete evaporation of the solvent, the polymer composite can be carefully removed from the casting chamber. The drawbacks of casted polymer composite films are crack formation, rigidness, and poor strength. In the melt intercalation process, the thermoplastic was melted over the *T_m_* and then mixed with nanofillers for proper dispersion. Later the solution was slowly cooled to form nanocomposites. The advantages of melt intercalation are no solvent is required, and also there is no chemical reaction. Extrusion is a relatively new method, where the plastic and cellulose fiber were compounded, later the extruded materials can be molded using compression molding, injection molding, etc.

## 11. Properties of Nanocellulose Reinforced Polymer Composites

Boufi et al. [145] isolated nanofibrillated cellulose (NFC) and cellulose nanocrystals (CNCs) from both the alfa plant and rachis of the date palm tree. For the preparation of CNC, the pre-treated fibers were acid hydrolyzed with H_2_SO_4_ and for the preparation of NFC, the pre-treated fibers were TEMPO-oxidized and later homogenized at high pressure. The morphology of CNC and NFC was dependent on the type of the source. The CNC and NFC of the date palm tree reported the highest aspect ratio compared to the alfa plant. The poly (styrene-co-butyl acrylate) composites were fabricated using the casting method. The DMA studies reported a significant reinforcing effect of the nanofiller on the storage modulus of the composites. A clear drop in modulus was visible for the neat polymer at the *T_g_*, but with the increasing addition of nanofiller, the drop in modulus at the *T_g_* was marginal due to the strong reinforcing effect of the nanofillers. It was observed that the reinforcing effect of NFC of date palm tree > CNC from date palm tree > NFC of alfa plant > CNC from alfa plant. On the other hand, the percolation threshold value follows the order NFC of date palm tree ˂ NFC of alfa plant ˂ CNC from date palm tree ˂ CNC from alfa plant. The transparency of the composite films was also tested. All the composites were transparent; however, the transparency was slightly reduced with the incorporation of nanofillers. Interestingly the scattering was more for the composites with smaller nanofiller (nanofiller from date palm tree) due to the increased percolation effect. Zielińska et al. [141] enzymatically hydrolyzed micrometric cellulose (Cel_A) with 20 μm particle size and micrometric cellulose (Cel_B) with 18 ± 3 μm particle size using cellulases. Cellulases from the microscopic fungus *trichoderma reesei ATCC 26,921* and Aspergillus sp. were used for the hydrolysis. The efficiency of the hydrolysis reaction was more when cellulases from *trichoderma reesei ATCC 26,921* was used, this results in cellulose in nanometer sale with low polydispersity. The obtained cellulose was further used to strengthen the PP matrix.

Rao et al. [21] fabricated polymer composites with a high amount of (60 to 90%) CNC in epoxide oligomer. The mixer was ultrasonicated and forms a gel. It was then 3D printed or cast and later thermally cured (resulting in crosslinking between the OH groups of cellulose and epoxide groups of epoxy monomers). Complex structures with cellulose nanocrystals with high strength, stiffness, toughness, and hardness can be developed by this approach. Somseemee and co-workers [146] studied the effect of UV radiation on the cellulose nanocrystal’s reinforcement on epoxidized natural rubber (ENR). The cellulose nanocrystals were extracted from Napier grass stems. The isolated cellulose nanocrystals and maleic anhydride-modified cellulose nanocrystals were used as the reinforcement for epoxidized natural rubber. The ENR and ENR composites were cured using ultraviolet irradiation. The maleic anhydride-grafted cellulose nanocrystals (5 wt%) modified epoxidized natural rubber showed the best tensile strength, modules, and hardness due to better reinforcement caused by the better interaction between modified cellulose nanocrystals and epoxidized natural rubber prompted by UV irradiation. 

Biopolymers are used more frequently in polymer composite technology, especially for composite structures that don’t need high strength. This is because the strength of biodegradable plastic is less than traditional plastics, another disadvantage is their high cost. One way to improve the strength of biodegradable plastic is the use of nanocellulose as a reinforcement. Ghasemi and co-workers [147] processed PLA with nanocellulose along with the compatibilizer maleated PLA (PLA-g-MA) through melt mixing and extrusion and the test specimens were prepared by injection molding. The composition of maleated PLA was 5 wt%, while that of the cellulose nanofibers was 3 and 5 wt%. Among the composites, PLA/CNF5/PLA-g-MA5 exhibited maximum HDT, maximum improvement (131%) in impact strength, maximum tensile strength (138% improvement), and a 40% improvement in Young’s modulus when compared to neat PLA. 

Cellulose waste is a material with great technological and economic importance, and it can be obtained from agricultural resources and from industrial wastes. de Souza, et al. [148] in their study, highlight the isolation of nanocellulose from both cotton waste and industrial paper wastes using acid hydrolysis. The cotton waste (10 wt%), industrial paper wastes (3 wt%), and isolated nanocellulose from cotton waste (10 wt%) and isolated nanocellulose from industrial paper wastes (3 wt%) were incorporated in the PLA matrix as reinforcement. The study revealed an improvement in tensile strength and elongation at break with the incorporation of nanocellulose. The increase in the properties was due to the better distribution of the filler and due to the chemical bonding of the OH group of nanocellulose and the CO group of the PLA matrix. Lamm et al. [149] explored the application of chitin as a dual-bonding filler in PLA/nanocellulose composites. The study reported that on one hand, chitosan formed hydrogen bonds with cellulose, and on the other hand, it formed amide bonds with poly(lactic acid) thus improving the interfacial interaction between the PLA and nanocellulose, and improving significantly the mechanical properties with a small amount of chitin (2.5 wt%).

Sultana et al. [150] extracted nanocellulose from betel nut husk fiber using H_2_SO_4_ and ultrasonic treatment. The characteristics of the nanocellulose such as chemical constituent (∞- cellulose 51.08%, hemicellulose 14.87%, lignin 14.87%, pectin 0.92%, and ash content 7.69%), structure (characteristic peaks of nanocellulose were observed from the FTIR), morphology (SEM) and particle size (117.6 nm) were measured. Further, nanocellulose-modified polyvinyl alcohol (PVA) composites were fabricated using the solvent casting method. Water is used as the solvent for making composites. The thermal stability of PVA/nanocellulose composites reported improved degradation temperature. The increased thermal stability was due to the hydrogen bonding between the cellulose and PVA. The tensile strength and elongation at break of the composites were also reported. It was observed that the composites with 2 wt% nanocellulose showed improved tensile strength followed by a decrease at a higher percentage of the nanocellulose. This was due to the aggregation of nanocellulose at higher concentrations. On the other hand, the elongation at break of PVA gradually reduced with the incorporation of nanocellulose. The gradual reduction in elongation at break was due to the restricted movement of polymer chains in the presence of the nanofillers.

Tang and Liu [151] prepared PVA composites with a non-transparent electron-spun cellulose nanofibrous mat. The mechanical properties and transparency were evaluated. The PVA composites with 40 wt% cellulose nanofibrous mat showed the best tensile strength and modulus, with more than 75% transparency. The high number of OH groups in nanocellulose mats and PVA was responsible for the improved mechanical properties and transparency. In another study, Gong et al. [152] reported improvement in tensile strength, tensile modulus, storage modulus, and improved creep resistance of polyvinyl acetate with the incorporation of 10 wt% nanocellulose.

Ethylene vinyl alcohol is known for its gas barrier properties, but it has poor moisture resistance that limits its application in food packaging. Nuruddin et al. [153] developed ethylene vinyl alcohol/cellulose nanocrystal composites modified with food-grade compatibilizer (monolaurin) by melt compounding process for potential food packaging applications. It was observed that the composites modified with monolaurin showed excellent compatibility with improved thermo-mechanical and moisture barrier properties. Takagi and Asano [154] studied the effect of nanocellulose fibers on the flexural properties of the starch matrix at different experimental conditions. The composites were prepared by hot pressing at different pressure (0 to 50 MPa) at 140 °C. The flexural modulus and flexural strength increase with increasing molding pressure. The increase in mechanical properties was due to the increased density of the composites at higher mold pressure. The optimum mold pressure used was 50 MPa. 

## 12. Applications of Cellulose Fiber Reinforced Polymer Composites

Cellulosic fiber-reinforced polymer composites were employed in plectra of applications such as in automobiles, aircraft, packaging, medical implant, electronics, building materials, etc. Some of the important studies on the application of cellulosic fiber-reinforced composites are discussed in this section. In an interesting work, Soni, Asoh, and Uyama [155] fabricated starch/cellulose nanofiber composites that are water insoluble. Here TEMPO-oxidized cellulose nanofiber was incorporated in three different modified starch (hydroxypropyl starch, acetyl starch, and acetyl oxidized starch), and the films were prepared by casting method. The TEMPO-oxidized cellulose nanofiber/hydroxypropyl starch (HPS) composite reported a significantly lower swelling ratio, the highest tensile modulus, and tensile strength, with good transparency. The exceptional performance of the TEMPO-oxidized cellulose nanofiber/hydroxypropyl starch (HPS) composite was due to the formation of hemiacetal bonds between the component polymers, because of the presence of the high number of OH groups in hydroxypropyl starch. The prepared films can be used as an alternative to single-use plastics.

**Figure 14:**
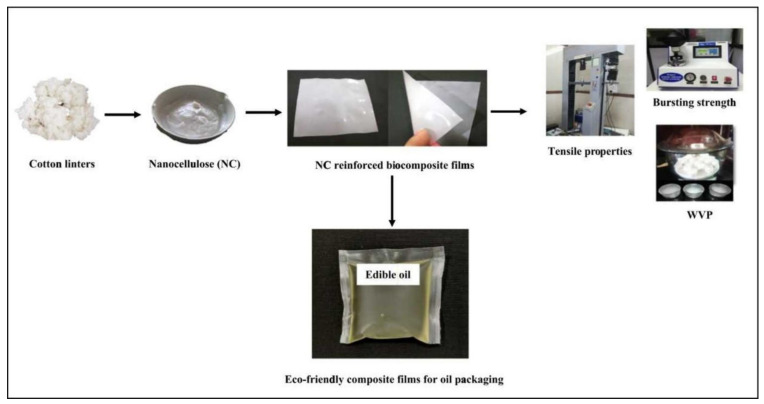
The schematic of the fabrication of eco-friendly composite films for oil packaging. Reprinted with permission from Ref. [158]. License Number: 5413161013792.

Polymer foams find applications in seat cushions, sound insulation materials, packaging, construction and building applications, electronic applications, automotive, and aerospace applications [156]. Ito et al. [157] fabricated PP/cellulose nanofiber composite foams and studied the effect of cellulose nanofiber content on the bubble size. PP have lower viscosity; therefore, they have a higher melt flow index, and hence bubbles undergo coalescence which results in a larger cellular structure. The rheology studies of the foam showed a gradual increase in storage modulus (G’) with filler content. At high nanofiber content (above 30 wt%) the G’ is independent of the frequency. The cell diameter of the foam is reduced with the fiber content, while the number density of the bubbles was increased. Coming to the mechanical properties, the composites with 30% fiber content showed a 1.8-fold increase in flexural modulus, an appreciable increase in flexural strength, and relative density (from 0.9 to 1.05). Food packaging comprises around 60% of the plastic used in the entire plastic industry. Therefore, recently many research works are undergoing on biodegradable plastics such as PLA, chitosan, starch, etc., in the food packaging industry. Patil et al. [158] reported modified starch films as an alternative material for storing edible oil (Figure 14). The researchers isolated nanocellulose from cotton linters, and later blended it with glycerol and polyvinyl alcohol, the films were prepared by using the solvent casting method. The authors reported improved mechanical and barrier properties for the composite films and the films/pouch were good enough to keep the oil for three months without compromising the oil quality. 

Wu and co-workers [159] reviewed the effect of nanocellulose on the gas barrier properties (oxygen, CO_2_, N_2_, water vapor, and air) of the different polymer composites. The authors highlighted that the incorporation of nanocellulose improved the gas barrier properties of different polymers. However, the gas barrier properties of the composites are badly affected in highly humid conditions due to the hydrophilicity of nanocellulose. This can be overcome to some extent by the addition of clay, or chitosan along with nanocellulose. The other method includes chemical modification of nanocellulose, for example, carboxylation, acid lauryl modification, etc. The modification improves the dispersion of the nanocellulose in the matrix and enhances the interfacial interaction between the nanocellulose and polymer. The mechanism of gas barrier properties is the tortuous path created by the nanocellulose. The evenly distributed nanocellulose caused the air or gas to pass through long distances and hence increased gas barrier properties. 

## 13. Conclusions

The importance of biodegradable materials has initiated the development of eco-friendly composites. Due to this reason, the extraction of cellulose from different sources such as plants, animals, bacteria, and algae has gained considerable attention among researchers. For the successful extraction of cellulose, several methods have been adopted by combining both chemical and mechanical treatments. The structure and the properties of the cellulose depend upon the source, and methods adopted for the extraction. Cellulose can exist in different forms such as cellulose nanocrystals, cellulose nanofibrils, and microcrystalline cellulose based on their dimension and appearance. Nanocellulose has potential applications in composites, tissue engineering, nanomedicine, biosensors, biodegradable polymers, power storage, and water treatment. The hydrophilic nature and the poor interfacial adhesion limit the use of cellulose in the composite industry. This can be overcome by chemical treatments, the addition of clay, chitosan, etc. Thus, making it an effective reinforcing filler in polymer matrices. The cellulosic fiber-reinforced polymer composites are employed in plectra of applications such as in automobiles, aircraft, construction and building, food packaging, construction and building, and water purification. In the coming years, nanocellulose will be the fundamental material for science and technology applications.

## Data Availability

Not applicable.
